# 4-(Dimethyl­amino)­benzaldehyde–2,4-di­nitro­aniline (1/1)

**DOI:** 10.1107/S160053681102232X

**Published:** 2011-06-18

**Authors:** Ruitao Zhu, Haoyang Li, Yuewen Zhang

**Affiliations:** a Department of Chemistry, Taiyuan Normal University, Taiyuan 030031, People’s Republic of China

## Abstract

The asymmetric unit of the title compound, C_9_H_11_NO·C_6_H_5_N_3_O_4_, contains two independent mol­ecules each of 4-(dimethyl­amino)­benzaldehyde and 2,4-dinitro­aniline. In the crystal, the components are linked by inter­molecular N—H⋯O hydrogen bonds to form one-dimensional chains along [10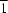]. Intra­molecular N—H⋯O hydrogen bonds are also present.

## Related literature

For related structures, see: Nesterov *et al.* (2000 ▶); Weber & Sheldrick (1981 ▶). For standard bond-length data, see: Allen *et al.* (1987 ▶).
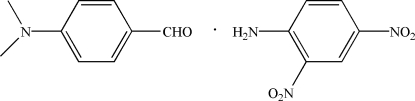


## Experimental

### 

#### Crystal data

C_9_H_11_NO·C_6_H_5_N_3_O_4_
*M* *_r_* = 332.32
Monoclinic, 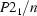

*a* = 18.7512 (18) Å
*b* = 7.3182 (6) Å
*c* = 24.338 (2) Å
β = 109.493 (1)°
*V* = 3148.4 (5) Å^3^
*Z* = 8
Mo *K*α radiation
μ = 0.11 mm^−1^
*T* = 298 K
0.48 × 0.18 × 0.11 mm

#### Data collection

Bruker SMART CCD diffractometer
Absorption correction: multi-scan (*SADABS*; Bruker, 2007 ▶) *T*_min_ = 0.950, *T*_max_ = 0.988
15396 measured reflections
5547 independent reflections
2303 reflections with *I* > 2σ(*I*)
*R*_int_ = 0.060

#### Refinement

*R*[*F* ^2^ > 2σ(*F* ^2^)] = 0.048
*wR*(*F* ^2^) = 0.087
*S* = 1.02
5547 reflections
433 parameters
6 restraints
H-atom parameters constrained
Δρ_max_ = 0.17 e Å^−3^
Δρ_min_ = −0.20 e Å^−3^

### 

Data collection: *SMART* (Bruker, 2007 ▶); cell refinement: *SAINT* (Bruker, 2007 ▶); data reduction: *SAINT*; program(s) used to solve structure: *SHELXS97* (Sheldrick, 2008 ▶); program(s) used to refine structure: *SHELXL97* (Sheldrick, 2008 ▶); molecular graphics: *SHELXTL* (Sheldrick, 2008 ▶) and *PLATON* (Spek, 2009 ▶); software used to prepare material for publication: *SHELXTL*.

## Supplementary Material

Crystal structure: contains datablock(s) I, global. DOI: 10.1107/S160053681102232X/lh5267sup1.cif

Structure factors: contains datablock(s) I. DOI: 10.1107/S160053681102232X/lh5267Isup2.hkl

Supplementary material file. DOI: 10.1107/S160053681102232X/lh5267Isup3.cml

Additional supplementary materials:  crystallographic information; 3D view; checkCIF report

## Figures and Tables

**Table 1:** Hydrogen-bond geometry (Å, °)

*D*—H⋯*A*	*D*—H	H⋯*A*	*D*⋯*A*	*D*—H⋯*A*
N1—H1*A*⋯O10^i^	0.86	2.18	3.010 (3)	163
N1—H1*B*⋯O1	0.86	2.03	2.640 (3)	127
N1—H1*B*⋯O7^ii^	0.86	2.49	3.170 (3)	136
N4—H4*A*⋯O9^iii^	0.86	2.04	2.889 (3)	171
N4—H4*B*⋯O5	0.86	2.02	2.636 (3)	128
N4—H4*B*⋯O3^iii^	0.86	2.42	3.047 (3)	130

Symmetry codes: (i) 
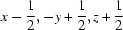
; (ii) 
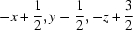
; (iii) 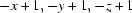.

**Fig. 1.**
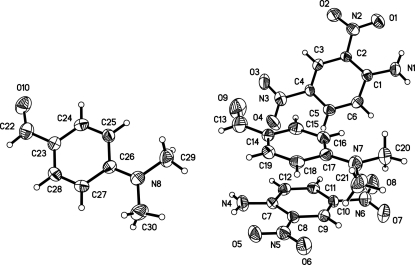
The molecular structure of the title compound with displacement ellipsoids are drawn at the 30% probability level.

**Fig. 2.**
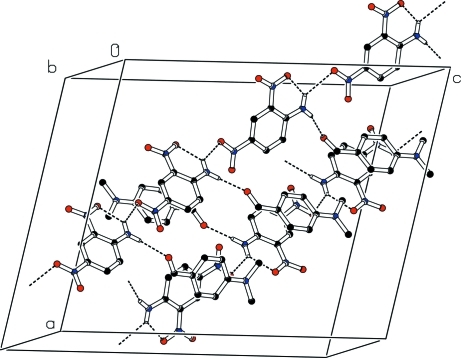
Part of the crystal structure of (I) with hydrogen bonds drawn as dashed lines. Only H atoms involved in hydrogen bonds are shown.

## supplementary crystallographic information

### Comment

The crystal structures of some molecular complexes of 2,4-dinitroaniline have already been published (Nesterov *et al.*, 2000; Weber & Sheldrick, 1981). In this paper, we present the crystal structure of the title compound (I).

The molecular structure of (I) is shown in Fig. 1. The bond lengths (Allen *et al.*, 1987) and angles are normal . The asymmetric unit contains two independent molecules of 4-(dimethylamino)benzaldehyde and two indpendent molecules of 2,4-Dinitroaniline. In the crystal, the components are linked by intermolecular N—H···O hydrogen bonds to form one-dimensional chains along [101].

### Experimental

A mixture of 4-(dimethylamino)benzaldehyde (0.75 g, 5 mmol) and 2,4-dinitroaniline (0.92 g, 5 mmol) were refluxed in ethanol (50 ml) for 30 min and rotary evaporated. Recrystallization from ethanol solution produced the crystals of the title compound.

### Refinement

H atoms were placed in idealized positions and allowed to ride on their respective parent atoms, with C—H = 0.93–0.96 Å, N—H = 0.86Å and *U*_iso_(H)= 1.2*U*_eq_(C,N) or 1.5*U*_eq_(C_methyl_).

### Crystal data

**Table:**

C_9_H_11_NO·C_6_H_5_N_3_O_4_	*F*(000) = 1392
*M**_r_* = 332.32	*D*_x_ = 1.402 Mg m^−^^3^
Monoclinic, *P*2_1_/*n*	Mo *K*α radiation, λ = 0.71073 Å
Hall symbol: -P 2yn	Cell parameters from 1665 reflections
*a* = 18.7512 (18) Å	θ = 2.5–20.6°
*b* = 7.3182 (6) Å	µ = 0.11 mm^−^^1^
*c* = 24.338 (2) Å	*T* = 298 K
β = 109.493 (1)°	Flake, colorless
*V* = 3148.4 (5) Å^3^	0.48 × 0.18 × 0.11 mm
*Z* = 8	

### Data collection

**Table:**

Bruker SMART CCD diffractometer	5547 independent reflections
Radiation source: fine-focus sealed tube	2303 reflections with *I* > 2σ(*I*)
graphite	*R*_int_ = 0.060
φ and ω scans	θ_max_ = 25.0°, θ_min_ = 2.4°
Absorption correction: multi-scan (*SADABS*; Bruker, 2007)	*h* = −22→16
*T*_min_ = 0.950, *T*_max_ = 0.988	*k* = −8→8
15396 measured reflections	*l* = −27→28

### Refinement

**Table:**

Refinement on *F*^2^	Primary atom site location: structure-invariant direct methods
Least-squares matrix: full	Secondary atom site location: difference Fourier map
*R*[*F*^2^ > 2σ(*F*^2^)] = 0.048	Hydrogen site location: inferred from neighbouring sites
*wR*(*F*^2^) = 0.087	H-atom parameters constrained
*S* = 1.02	*w* = 1/[σ^2^(*F*_o_^2^) + (0.0105*P*)^2^] where *P* = (*F*_o_^2^ + 2*F*_c_^2^)/3
5547 reflections	(Δ/σ)_max_ < 0.001
433 parameters	Δρ_max_ = 0.17 e Å^−^^3^
6 restraints	Δρ_min_ = −0.20 e Å^−^^3^

### Special details

**Table:**

Geometry. All e.s.d.'s (except the e.s.d. in the dihedral angle between two l.s. planes) are estimated using the full covariance matrix. The cell e.s.d.'s are taken into account individually in the estimation of e.s.d.'s in distances, angles and torsion angles; correlations between e.s.d.'s in cell parameters are only used when they are defined by crystal symmetry. An approximate (isotropic) treatment of cell e.s.d.'s is used for estimating e.s.d.'s involving l.s. planes.
Refinement. Refinement of *F*^2^ against ALL reflections. The weighted *R*-factor *wR* and goodness of fit *S* are based on *F*^2^, conventional *R*-factors *R* are based on *F*, with *F* set to zero for negative *F*^2^. The threshold expression of *F*^2^ > σ(*F*^2^) is used only for calculating *R*-factors(gt) *etc*. and is not relevant to the choice of reflections for refinement. *R*-factors based on *F*^2^ are statistically about twice as large as those based on *F*, and *R*- factors based on ALL data will be even larger.

### Fractional atomic coordinates and isotropic or equivalent isotropic displacement parameters (Å^2^)

**Table:**

	*x*	*y*	*z*	*U*_iso_*/*U*_eq_	
N1	0.13405 (12)	0.4177 (3)	0.64855 (9)	0.0680 (7)	
H1A	0.1570	0.4459	0.6844	0.082*	
H1B	0.0892	0.3723	0.6382	0.082*	
N2	0.06120 (14)	0.3253 (3)	0.52459 (12)	0.0627 (7)	
N3	0.28629 (16)	0.5458 (4)	0.49136 (12)	0.0659 (8)	
N4	0.62137 (12)	0.6263 (3)	0.60594 (9)	0.0730 (8)	
H4A	0.5991	0.6520	0.5698	0.088*	
H4B	0.6661	0.5802	0.6172	0.088*	
N5	0.69136 (14)	0.5410 (3)	0.73034 (12)	0.0640 (7)	
N6	0.46369 (16)	0.7627 (3)	0.75848 (12)	0.0644 (7)	
N7	0.54203 (14)	0.1901 (3)	0.78750 (11)	0.0702 (8)	
N8	0.79608 (13)	0.0474 (3)	0.53617 (10)	0.0672 (7)	
O1	0.02442 (11)	0.2921 (3)	0.55713 (9)	0.0863 (7)	
O2	0.03664 (10)	0.2926 (3)	0.47188 (9)	0.0859 (7)	
O3	0.25494 (12)	0.5097 (3)	0.43926 (10)	0.0856 (7)	
O4	0.34929 (12)	0.6141 (3)	0.51053 (9)	0.0911 (8)	
O5	0.72847 (10)	0.4992 (3)	0.69840 (9)	0.0838 (7)	
O6	0.71548 (10)	0.5136 (3)	0.78306 (9)	0.0864 (7)	
O7	0.49526 (11)	0.7428 (3)	0.81136 (9)	0.0842 (7)	
O8	0.39780 (12)	0.8089 (3)	0.73743 (9)	0.1019 (9)	
O9	0.45542 (14)	0.2464 (4)	0.51132 (9)	0.1171 (10)	
O10	0.72731 (12)	−0.0839 (3)	0.26313 (9)	0.0903 (8)	
C1	0.16792 (16)	0.4443 (4)	0.60916 (12)	0.0481 (8)	
C2	0.13580 (14)	0.4027 (3)	0.54922 (12)	0.0450 (7)	
C3	0.17543 (15)	0.4359 (3)	0.51131 (11)	0.0490 (8)	
H3	0.1537	0.4080	0.4719	0.059*	
C4	0.24568 (16)	0.5087 (4)	0.53145 (13)	0.0477 (7)	
C5	0.27963 (15)	0.5507 (3)	0.59022 (13)	0.0516 (8)	
H5	0.3281	0.6002	0.6037	0.062*	
C6	0.24120 (15)	0.5186 (4)	0.62736 (12)	0.0520 (8)	
H6	0.2642	0.5469	0.6666	0.062*	
C7	0.58660 (16)	0.6579 (4)	0.64448 (12)	0.0491 (7)	
C8	0.61777 (14)	0.6216 (4)	0.70481 (11)	0.0472 (7)	
C9	0.57746 (15)	0.6585 (4)	0.74155 (11)	0.0511 (8)	
H9	0.5987	0.6345	0.7812	0.061*	
C10	0.50712 (16)	0.7295 (4)	0.72008 (12)	0.0463 (7)	
C11	0.47405 (15)	0.7694 (3)	0.66082 (12)	0.0507 (8)	
H11	0.4258	0.8197	0.6465	0.061*	
C12	0.51349 (16)	0.7334 (4)	0.62468 (12)	0.0535 (8)	
H12	0.4915	0.7596	0.5852	0.064*	
C13	0.5127 (2)	0.2137 (4)	0.55089 (14)	0.0854 (11)	
H13	0.5553	0.1856	0.5410	0.102*	
C14	0.52185 (17)	0.2138 (4)	0.61208 (12)	0.0536 (8)	
C15	0.46240 (16)	0.2653 (4)	0.63065 (13)	0.0571 (8)	
H15	0.4171	0.3036	0.6034	0.069*	
C16	0.46925 (15)	0.2606 (3)	0.68821 (13)	0.0536 (8)	
H16	0.4288	0.2980	0.6995	0.064*	
C17	0.53573 (17)	0.2010 (4)	0.73066 (13)	0.0516 (8)	
C18	0.59579 (15)	0.1494 (4)	0.71130 (12)	0.0563 (8)	
H18	0.6411	0.1096	0.7382	0.068*	
C19	0.58822 (15)	0.1569 (4)	0.65354 (13)	0.0590 (8)	
H19	0.6288	0.1229	0.6419	0.071*	
C20	0.48038 (17)	0.2435 (4)	0.80759 (12)	0.0838 (11)	
H20A	0.4740	0.3736	0.8045	0.126*	
H20B	0.4918	0.2071	0.8475	0.126*	
H20C	0.4346	0.1849	0.7840	0.126*	
C21	0.61157 (16)	0.1308 (4)	0.83177 (11)	0.0912 (11)	
H21A	0.6202	0.0040	0.8260	0.137*	
H21B	0.6073	0.1472	0.8697	0.137*	
H21C	0.6532	0.2019	0.8288	0.137*	
C22	0.77930 (18)	−0.0195 (4)	0.30214 (14)	0.0732 (10)	
H22	0.8208	0.0196	0.2925	0.088*	
C23	0.78384 (17)	0.0032 (4)	0.36245 (13)	0.0523 (8)	
C24	0.72267 (15)	−0.0435 (3)	0.37970 (13)	0.0542 (8)	
H24	0.6786	−0.0873	0.3521	0.065*	
C25	0.72604 (15)	−0.0264 (4)	0.43635 (13)	0.0534 (8)	
H25	0.6838	−0.0552	0.4466	0.064*	
C26	0.79240 (16)	0.0341 (4)	0.47947 (12)	0.0495 (8)	
C27	0.85369 (15)	0.0821 (4)	0.46156 (12)	0.0547 (8)	
H27	0.8981	0.1253	0.4888	0.066*	
C28	0.84891 (15)	0.0661 (4)	0.40456 (13)	0.0574 (8)	
H28	0.8904	0.0982	0.3938	0.069*	
C29	0.73431 (16)	−0.0077 (4)	0.55566 (12)	0.0807 (10)	
H29A	0.6893	0.0574	0.5339	0.121*	
H29B	0.7468	0.0196	0.5964	0.121*	
H29C	0.7259	−0.1366	0.5496	0.121*	
C30	0.86512 (15)	0.1111 (4)	0.58117 (11)	0.0862 (11)	
H30A	0.9054	0.0262	0.5846	0.129*	
H30B	0.8564	0.1195	0.6178	0.129*	
H30C	0.8787	0.2291	0.5706	0.129*	

### Atomic displacement parameters (Å^2^)

**Table:**

	*U*^11^	*U*^22^	*U*^33^	*U*^12^	*U*^13^	*U*^23^
N1	0.0661 (17)	0.091 (2)	0.0563 (16)	−0.0038 (14)	0.0327 (14)	−0.0031 (14)
N2	0.0510 (15)	0.0666 (19)	0.0671 (16)	−0.0032 (14)	0.0151 (15)	0.0009 (15)
N3	0.066 (2)	0.070 (2)	0.073 (2)	0.0053 (16)	0.0392 (16)	0.0088 (16)
N4	0.0634 (17)	0.106 (2)	0.0558 (17)	0.0032 (15)	0.0291 (14)	0.0089 (15)
N5	0.0491 (16)	0.074 (2)	0.066 (2)	0.0013 (13)	0.0150 (15)	0.0039 (16)
N6	0.066 (2)	0.069 (2)	0.064 (2)	0.0077 (16)	0.0308 (18)	−0.0010 (15)
N7	0.0644 (19)	0.092 (2)	0.0545 (18)	0.0014 (15)	0.0208 (15)	0.0027 (16)
N8	0.0635 (18)	0.088 (2)	0.0477 (17)	−0.0110 (15)	0.0159 (15)	−0.0084 (15)
O1	0.0620 (15)	0.111 (2)	0.0961 (18)	−0.0151 (13)	0.0400 (13)	−0.0025 (14)
O2	0.0702 (14)	0.113 (2)	0.0612 (13)	−0.0207 (12)	0.0047 (11)	−0.0049 (13)
O3	0.0930 (17)	0.113 (2)	0.0658 (16)	−0.0017 (14)	0.0470 (14)	−0.0052 (14)
O4	0.0623 (15)	0.118 (2)	0.1038 (18)	−0.0139 (14)	0.0420 (14)	0.0113 (15)
O5	0.0511 (14)	0.126 (2)	0.0819 (17)	0.0089 (13)	0.0323 (12)	−0.0041 (14)
O6	0.0655 (15)	0.129 (2)	0.0580 (15)	0.0167 (13)	0.0118 (12)	0.0150 (14)
O7	0.0899 (17)	0.113 (2)	0.0571 (15)	0.0218 (13)	0.0342 (13)	0.0084 (13)
O8	0.0687 (16)	0.157 (2)	0.0879 (18)	0.0381 (16)	0.0369 (14)	0.0113 (15)
O9	0.128 (2)	0.162 (3)	0.0519 (15)	0.0101 (18)	0.0164 (14)	0.0233 (16)
O10	0.1016 (18)	0.112 (2)	0.0499 (14)	0.0068 (15)	0.0155 (12)	−0.0085 (13)
C1	0.051 (2)	0.046 (2)	0.053 (2)	0.0062 (15)	0.0244 (16)	0.0027 (15)
C2	0.0408 (15)	0.045 (2)	0.0504 (18)	0.0028 (13)	0.0170 (12)	0.0015 (14)
C3	0.057 (2)	0.048 (2)	0.0405 (18)	0.0030 (16)	0.0148 (16)	−0.0024 (14)
C4	0.051 (2)	0.047 (2)	0.049 (2)	0.0000 (16)	0.0227 (14)	0.0008 (15)
C5	0.0431 (18)	0.049 (2)	0.062 (2)	0.0000 (15)	0.0164 (17)	−0.0012 (16)
C6	0.054 (2)	0.055 (2)	0.0433 (19)	0.0025 (16)	0.0102 (16)	−0.0031 (15)
C7	0.0493 (19)	0.050 (2)	0.053 (2)	−0.0094 (16)	0.0245 (16)	−0.0015 (16)
C8	0.0407 (16)	0.057 (2)	0.0443 (18)	−0.0019 (14)	0.0142 (15)	0.0034 (15)
C9	0.0520 (19)	0.058 (2)	0.0426 (18)	−0.0055 (16)	0.0141 (16)	0.0020 (15)
C10	0.051 (2)	0.047 (2)	0.048 (2)	−0.0009 (15)	0.0255 (16)	−0.0028 (15)
C11	0.0445 (18)	0.050 (2)	0.054 (2)	−0.0010 (14)	0.0113 (16)	0.0024 (16)
C12	0.056 (2)	0.058 (2)	0.0407 (19)	−0.0002 (16)	0.0082 (16)	0.0032 (15)
C13	0.104 (3)	0.095 (3)	0.0597 (19)	−0.008 (2)	0.031 (2)	0.006 (2)
C14	0.054 (2)	0.055 (2)	0.0513 (17)	−0.0061 (17)	0.0174 (18)	0.0000 (16)
C15	0.049 (2)	0.054 (2)	0.059 (2)	−0.0011 (16)	0.0040 (17)	0.0079 (16)
C16	0.0452 (19)	0.055 (2)	0.062 (2)	−0.0006 (15)	0.0197 (17)	−0.0003 (17)
C17	0.056 (2)	0.049 (2)	0.048 (2)	−0.0065 (16)	0.0157 (18)	0.0015 (16)
C18	0.0452 (19)	0.060 (2)	0.057 (2)	−0.0002 (16)	0.0069 (16)	0.0016 (17)
C19	0.050 (2)	0.066 (2)	0.064 (2)	−0.0027 (17)	0.0236 (17)	−0.0055 (18)
C20	0.096 (3)	0.099 (3)	0.072 (2)	−0.012 (2)	0.048 (2)	−0.0015 (19)
C21	0.097 (3)	0.119 (3)	0.048 (2)	0.005 (2)	0.0117 (19)	0.005 (2)
C22	0.086 (3)	0.075 (3)	0.062 (3)	0.008 (2)	0.029 (2)	0.006 (2)
C23	0.058 (2)	0.051 (2)	0.048 (2)	0.0055 (17)	0.0167 (17)	0.0034 (15)
C24	0.053 (2)	0.048 (2)	0.054 (2)	−0.0001 (15)	0.0065 (16)	−0.0027 (16)
C25	0.049 (2)	0.056 (2)	0.058 (2)	0.0012 (15)	0.0215 (17)	0.0003 (16)
C26	0.054 (2)	0.047 (2)	0.0440 (19)	0.0023 (16)	0.0107 (17)	0.0012 (15)
C27	0.0470 (19)	0.054 (2)	0.057 (2)	−0.0048 (15)	0.0098 (16)	−0.0022 (16)
C28	0.054 (2)	0.056 (2)	0.066 (2)	−0.0014 (16)	0.0256 (18)	0.0063 (18)
C29	0.097 (3)	0.098 (3)	0.058 (2)	0.000 (2)	0.040 (2)	0.0065 (19)
C30	0.089 (2)	0.112 (3)	0.046 (2)	−0.012 (2)	0.0060 (18)	−0.0040 (19)

### Geometric parameters (Å, °)

**Table:**

N1—C1	1.329 (3)	C10—C11	1.398 (3)
N1—H1A	0.8600	C11—C12	1.350 (3)
N1—H1B	0.8600	C11—H11	0.9300
N2—O2	1.233 (3)	C12—H12	0.9300
N2—O1	1.236 (3)	C13—C14	1.441 (4)
N2—C2	1.441 (3)	C13—H13	0.9300
N3—O4	1.223 (3)	C14—C19	1.379 (3)
N3—O3	1.235 (3)	C14—C15	1.387 (3)
N3—C4	1.450 (3)	C15—C16	1.364 (3)
N4—C7	1.329 (3)	C15—H15	0.9300
N4—H4A	0.8600	C16—C17	1.397 (3)
N4—H4B	0.8600	C16—H16	0.9300
N5—O6	1.226 (3)	C17—C18	1.410 (3)
N5—O5	1.242 (3)	C18—C19	1.366 (3)
N5—C8	1.437 (3)	C18—H18	0.9300
N6—O8	1.217 (2)	C19—H19	0.9300
N6—O7	1.233 (2)	C20—H20A	0.9600
N6—C10	1.450 (3)	C20—H20B	0.9600
N7—C17	1.351 (3)	C20—H20C	0.9600
N7—C20	1.451 (3)	C21—H21A	0.9600
N7—C21	1.453 (3)	C21—H21B	0.9600
N8—C26	1.362 (3)	C21—H21C	0.9600
N8—C29	1.448 (3)	C22—C23	1.451 (4)
N8—C30	1.465 (3)	C22—H22	0.9300
O9—C13	1.204 (3)	C23—C28	1.384 (3)
O10—C22	1.207 (3)	C23—C24	1.389 (3)
C1—C6	1.405 (3)	C24—C25	1.365 (3)
C1—C2	1.413 (3)	C24—H24	0.9300
C2—C3	1.386 (3)	C25—C26	1.405 (3)
C3—C4	1.352 (3)	C25—H25	0.9300
C3—H3	0.9300	C26—C27	1.403 (3)
C4—C5	1.392 (3)	C27—C28	1.365 (3)
C5—C6	1.352 (3)	C27—H27	0.9300
C5—H5	0.9300	C28—H28	0.9300
C6—H6	0.9300	C29—H29A	0.9600
C7—C12	1.406 (3)	C29—H29B	0.9600
C7—C8	1.413 (3)	C29—H29C	0.9600
C8—C9	1.377 (3)	C30—H30A	0.9600
C9—C10	1.350 (3)	C30—H30B	0.9600
C9—H9	0.9300	C30—H30C	0.9600
			
C1—N1—H1A	120.0	C19—C14—C15	118.1 (3)
C1—N1—H1B	120.0	C19—C14—C13	121.5 (3)
H1A—N1—H1B	120.0	C15—C14—C13	120.4 (3)
O2—N2—O1	122.3 (3)	C16—C15—C14	121.2 (3)
O2—N2—C2	118.8 (3)	C16—C15—H15	119.4
O1—N2—C2	118.9 (3)	C14—C15—H15	119.4
O4—N3—O3	123.2 (3)	C15—C16—C17	121.5 (3)
O4—N3—C4	118.5 (3)	C15—C16—H16	119.3
O3—N3—C4	118.3 (3)	C17—C16—H16	119.3
C7—N4—H4A	120.0	N7—C17—C16	121.8 (3)
C7—N4—H4B	120.0	N7—C17—C18	121.3 (3)
H4A—N4—H4B	120.0	C16—C17—C18	116.9 (3)
O6—N5—O5	121.8 (3)	C19—C18—C17	120.9 (3)
O6—N5—C8	119.0 (3)	C19—C18—H18	119.6
O5—N5—C8	119.2 (3)	C17—C18—H18	119.6
O8—N6—O7	122.6 (3)	C18—C19—C14	121.6 (3)
O8—N6—C10	119.0 (3)	C18—C19—H19	119.2
O7—N6—C10	118.4 (3)	C14—C19—H19	119.2
C17—N7—C20	121.5 (3)	N7—C20—H20A	109.5
C17—N7—C21	121.8 (3)	N7—C20—H20B	109.5
C20—N7—C21	116.7 (3)	H20A—C20—H20B	109.5
C26—N8—C29	122.4 (3)	N7—C20—H20C	109.5
C26—N8—C30	120.9 (2)	H20A—C20—H20C	109.5
C29—N8—C30	116.6 (2)	H20B—C20—H20C	109.5
N1—C1—C6	118.8 (3)	N7—C21—H21A	109.5
N1—C1—C2	124.9 (3)	N7—C21—H21B	109.5
C6—C1—C2	116.3 (3)	H21A—C21—H21B	109.5
C3—C2—C1	120.7 (3)	N7—C21—H21C	109.5
C3—C2—N2	117.0 (3)	H21A—C21—H21C	109.5
C1—C2—N2	122.3 (3)	H21B—C21—H21C	109.5
C4—C3—C2	120.1 (2)	O10—C22—C23	126.9 (3)
C4—C3—H3	119.9	O10—C22—H22	116.6
C2—C3—H3	119.9	C23—C22—H22	116.6
C3—C4—C5	121.0 (3)	C28—C23—C24	118.1 (3)
C3—C4—N3	119.8 (3)	C28—C23—C22	121.8 (3)
C5—C4—N3	119.2 (3)	C24—C23—C22	120.1 (3)
C6—C5—C4	119.1 (3)	C25—C24—C23	121.2 (3)
C6—C5—H5	120.5	C25—C24—H24	119.4
C4—C5—H5	120.5	C23—C24—H24	119.4
C5—C6—C1	122.7 (3)	C24—C25—C26	121.0 (3)
C5—C6—H6	118.7	C24—C25—H25	119.5
C1—C6—H6	118.7	C26—C25—H25	119.5
N4—C7—C12	118.6 (3)	N8—C26—C27	121.8 (3)
N4—C7—C8	124.8 (3)	N8—C26—C25	120.8 (3)
C12—C7—C8	116.6 (3)	C27—C26—C25	117.4 (3)
C9—C8—C7	120.8 (3)	C28—C27—C26	120.8 (3)
C9—C8—N5	117.1 (2)	C28—C27—H27	119.6
C7—C8—N5	122.1 (3)	C26—C27—H27	119.6
C10—C9—C8	120.1 (3)	C27—C28—C23	121.6 (3)
C10—C9—H9	120.0	C27—C28—H28	119.2
C8—C9—H9	120.0	C23—C28—H28	119.2
C9—C10—C11	121.3 (3)	N8—C29—H29A	109.5
C9—C10—N6	120.1 (3)	N8—C29—H29B	109.5
C11—C10—N6	118.6 (3)	H29A—C29—H29B	109.5
C12—C11—C10	118.7 (3)	N8—C29—H29C	109.5
C12—C11—H11	120.6	H29A—C29—H29C	109.5
C10—C11—H11	120.6	H29B—C29—H29C	109.5
C11—C12—C7	122.4 (3)	N8—C30—H30A	109.5
C11—C12—H12	118.8	N8—C30—H30B	109.5
C7—C12—H12	118.8	H30A—C30—H30B	109.5
O9—C13—C14	126.4 (4)	N8—C30—H30C	109.5
O9—C13—H13	116.8	H30A—C30—H30C	109.5
C14—C13—H13	116.8	H30B—C30—H30C	109.5
			
N1—C1—C2—C3	179.8 (2)	C9—C10—C11—C12	0.8 (4)
C6—C1—C2—C3	−0.4 (4)	N6—C10—C11—C12	−178.0 (2)
N1—C1—C2—N2	0.0 (4)	C10—C11—C12—C7	−0.2 (4)
C6—C1—C2—N2	179.8 (2)	N4—C7—C12—C11	179.8 (2)
O2—N2—C2—C3	0.1 (4)	C8—C7—C12—C11	−0.4 (4)
O1—N2—C2—C3	179.3 (2)	O9—C13—C14—C19	173.6 (3)
O2—N2—C2—C1	179.9 (3)	O9—C13—C14—C15	−4.0 (5)
O1—N2—C2—C1	−0.9 (4)	C19—C14—C15—C16	0.3 (4)
C1—C2—C3—C4	0.0 (4)	C13—C14—C15—C16	177.9 (3)
N2—C2—C3—C4	179.9 (2)	C14—C15—C16—C17	−1.2 (4)
C2—C3—C4—C5	0.3 (4)	C20—N7—C17—C16	−1.3 (4)
C2—C3—C4—N3	−179.0 (2)	C21—N7—C17—C16	−179.0 (3)
O4—N3—C4—C3	179.0 (3)	C20—N7—C17—C18	179.9 (3)
O3—N3—C4—C3	0.2 (4)	C21—N7—C17—C18	2.1 (4)
O4—N3—C4—C5	−0.4 (4)	C15—C16—C17—N7	−177.7 (3)
O3—N3—C4—C5	−179.2 (3)	C15—C16—C17—C18	1.2 (4)
C3—C4—C5—C6	−0.3 (4)	N7—C17—C18—C19	178.5 (3)
N3—C4—C5—C6	179.0 (2)	C16—C17—C18—C19	−0.4 (4)
C4—C5—C6—C1	−0.1 (4)	C17—C18—C19—C14	−0.4 (4)
N1—C1—C6—C5	−179.7 (2)	C15—C14—C19—C18	0.5 (4)
C2—C1—C6—C5	0.4 (4)	C13—C14—C19—C18	−177.1 (3)
N4—C7—C8—C9	−179.9 (2)	O10—C22—C23—C28	174.9 (3)
C12—C7—C8—C9	0.3 (4)	O10—C22—C23—C24	−3.2 (5)
N4—C7—C8—N5	−1.5 (4)	C28—C23—C24—C25	0.7 (4)
C12—C7—C8—N5	178.7 (2)	C22—C23—C24—C25	178.8 (2)
O6—N5—C8—C9	−1.9 (4)	C23—C24—C25—C26	−1.9 (4)
O5—N5—C8—C9	177.4 (3)	C29—N8—C26—C27	−177.7 (3)
O6—N5—C8—C7	179.6 (3)	C30—N8—C26—C27	−0.7 (4)
O5—N5—C8—C7	−1.1 (4)	C29—N8—C26—C25	3.2 (4)
C7—C8—C9—C10	0.3 (4)	C30—N8—C26—C25	−179.8 (2)
N5—C8—C9—C10	−178.2 (2)	C24—C25—C26—N8	−178.6 (3)
C8—C9—C10—C11	−0.9 (4)	C24—C25—C26—C27	2.2 (4)
C8—C9—C10—N6	177.9 (2)	N8—C26—C27—C28	179.4 (2)
O8—N6—C10—C9	−172.5 (3)	C25—C26—C27—C28	−1.4 (4)
O7—N6—C10—C9	7.5 (4)	C26—C27—C28—C23	0.3 (4)
O8—N6—C10—C11	6.3 (4)	C24—C23—C28—C27	0.1 (4)
O7—N6—C10—C11	−173.7 (3)	C22—C23—C28—C27	−178.0 (3)

### Hydrogen-bond geometry (Å, °)

**Table:**

*D*—H···*A*	*D*—H	H···*A*	*D*···*A*	*D*—H···*A*
N1—H1A···O10^i^	0.86	2.18	3.010 (3)	163
N1—H1B···O1	0.86	2.03	2.640 (3)	127
N1—H1B···O7^ii^	0.86	2.49	3.170 (3)	136
N4—H4A···O9^iii^	0.86	2.04	2.889 (3)	171
N4—H4B···O5	0.86	2.02	2.636 (3)	128
N4—H4B···O3^iii^	0.86	2.42	3.047 (3)	130

Symmetry codes: (i) *x*−1/2, −*y*+1/2, *z*+1/2; (ii) −*x*+1/2, *y*−1/2, −*z*+3/2; (iii) −*x*+1, −*y*+1, −*z*+1.
